# Active and passive arm movements do not change affective evaluation

**DOI:** 10.1371/journal.pone.0327881

**Published:** 2025-07-11

**Authors:** Áron Horváth, János Körmendi, Orsolya Drozdovszky, Vera Gál, Ferenc Köteles

**Affiliations:** 1 Institute of Psychology, Károli Gáspár University of the Reformed Church in Hungary, Budapest, Hungary; 2 Ádám György Psychophysiology Research Group, Budapest, Hungary; 3 Institute of Health Promotion and Sport Sciences, ELTE Eötvös Loránd University, Budapest, Hungary; University of Perugia: Universita degli Studi di Perugia, ITALY

## Abstract

Previous research showed that the activation of the arm extensor muscles is associated with a more negative attitude. In contrast, arm flexor muscle activation is associated with a more positive one. However, most of the studies tested these effects with isometric activation of the muscles. The current study was conducted to test if these effects occur with actual passive and active forearm movement (i.e., isotonic muscle activation). 56 university students participated in this preregistered experiment. The study design consisted of two within-subject factors: (1) type of motion (i.e., the arm of the participants was moved actively or passively), and (2) movement direction (i.e., the arm moved toward the body, away from the body, or remained at the middle position. Participants rated their preferences toward Chinese-like ideographs. Data was analysed with repeated measures (2x3) ANCOVAs with state and trait affectivity (i.e., the experience of positive and negative emotional states at the moment and the general tendency to experience them) as covariates. No significant type of motion and direction main effects, and interaction were found. In conclusion, slight isotonic and passive movements of the forearm do not change the affective evaluation of stimuli. Systematic manipulation of the parameters, most importantly the muscle effort needed to move the forearm, could shed more light on the background of the results.

## Introduction

Research on the relationship between motor behaviour, affective evaluation, and attitudes has a long history; it has its roots in the works of Charles Darwin and William James [1]. In terms of arm movements, activation of the flexor muscles of the elbow (biceps), which pull objects toward the body, is assumed to be associated with positive evaluation, whereas activation of the extensor (triceps) muscles, which push away undesired objects, is supposed to be associated with negative evaluation. The finding that activation of the respective muscles of the leg is not related to similar attitudinal changes gives further credit to the idea that manipulation with hands can play a role in the phenomenon [2].

Concerning the empirical literature, there are two main approaches to this topic. One line of research concentrates on how valenced (positive or negative) stimuli affect motor actions associated with approach and avoidance behaviour, such as arm flexion and extension, respectively. In the early study of Solarz [3], participants pulled cards with pleasant words faster toward themselves than cards with unpleasant words and pushed away cards with unpleasant words quicker than cards with pleasant words. In a similar vein, participants reacted faster when they evaluated positive words by pulling a lever and negative words by pushing a lever than when the pattern was the opposite [4]. Later studies refined the picture, concluding that the effect of stimuli does not necessarily depend on which muscle is contracted, but on the goal and label (i.e., the meaning) of the movement. Markman and Brendl [5] placed the names of their participants on the computer screen and instructed them to push and pull positive and negative words toward their names with a joystick. In this setting, participants were faster pushing (using extensor muscles) positive words and pulling negative words (using flexor muscles). Eder and Rothermund [6] found that labelling is essential for the effect to occur, as it was only present if the motor actions were labelled as toward and away, but not when the same movements were labelled downward and upward. Bamford and Wand [7] also showed that the consequence of the motor response is more important than flexor and extensor muscle activation. Empirical findings have been summarized in two recent meta-analyses [8,9]. Phaf and colleagues [9] found that an overall significant effect is present only if the instruction is explicit, meaning that pulling movements are interpreted as approach, and pushing movements as avoidance. Laham and colleagues [8] also showed that the effect is present only if the instruction is explicit; in addition, they identified important moderators. In more detail, the effect was larger when the stimuli were anger-related and consisted of faces (compared to words and pictorial stimuli). Also, vertical button presses facilitated the effect. Overall, these findings do not support the idea that valenced stimuli have a direct effect on the activation of specific (arm extensor and flexor) muscles, regardless of the context [8].

The other line of resources focuses on the effect of motor actions on the development of attitudes. According to the findings of Cacioppo and colleagues [2], initially neutral stimuli were evaluated more positively after becoming associated with isometric arm flexion, whereas arm extension caused a shift toward the opposite direction. Interestingly, no such changes in affective evaluation were observed during the periods of actual muscular effort. The authors concluded that “active motor processes or their sensory consequences can play a role in attitude development” [2]. In line with these findings, participants performing arm flexion generated more names of positively evaluated celebrities, whereas arm extension facilitated the generation of names toward whom they had a negative attitude [10]. In the study of Neumann and Strack [1] (Experiment 1) isometric arm flexion and extension were associated with a faster categorization of positive and negative adjectives, respectively. Cretenet and colleagues [11] refined the picture, showing that unilateral flexion on the right side (activating the left hemisphere that is linked to positive/approach orientation) and unilateral extension on the left side (activating the right hemisphere with negative/avoidance orientation) are associated with a more positive evaluation. These findings suggest that congruence between the motor program and the direction of evaluation might be more important than the motor program alone [12]. The importance of specific muscle activation was also questioned in this line of research, as Van Dessel and colleagues [13] found that imagination of movements is sufficient to evoke an effect. Woud and colleagues [14] showed that the effect may not work on valenced, but only on neutral stimuli. In their experiment, participants pulled or pushed faces away or closer with a joystick. Implicit attitudes were influenced only if the faces were neutral, but not when angry or smiling. Finally, Van Dessel and colleagues [15] found that the instruction might also be important. Telling participants that they would move toward a stimulus (self-agent instruction) produced stronger effects than the instruction that the stimulus would move toward them (stimulus-agent instruction).

Relying on these findings, researchers interested in psychological processes behind consuming assumed that approach/avoidance behaviour, triggered by activation of the upper arm muscles, might impact shopping choices. Empirical results showed that carrying a shopping basket (i.e., continuous arm flexion) led to more purchases of products with hedonistic value than moving a shopping cart (i.e., continuous arm extension) [16]. Also, pushing a shopping cart with flexed and extended arms increased and decreased the number of products put into the cart, respectively, in the study of Streicher and Estes [17]. Finally, changing the handles of the shopping cart led to more purchases for handles that were designed to activate the flexor muscles compared to generally used handles that activate the extensors [18].

The current study has been prepared in the framework of how motor actions affect the development of attitudes. On the reverse relationship (i.e., effect of valenced stimuli on movements), many studies consistently show the lack of direct effect [6]. Based on that, however, we cannot draw the same conclusion for the effect of motion on affective evaluation. The observation that imagined movements can alone cause changes in attitudes [13] also does not rule out this possibility, as imagined movements can activate similar brain areas than the movement actually executed (in more detail, see below) [19]. Also, in the majority of the aforementioned studies, static (i.e., isometric) activation of the upper arm flexor and extensor muscles was considered a stimulus that interacts with affective evaluation. In the widely used paradigm developed by Cacioppo and colleagues [2], participants were asked to place their palm(s) on a deck in front of them and push it downwards (isometric activation of extensors), or to put their palms under the deck and push it upwards (isometric activation of flexors). Similarly, isometric muscle activation was induced, via the classic method or carrying/moving a shopping basket/cart in studies on shopping preferences [16–18]. In other words, no actual forearm movement (i.e., change in the angle of the elbow joint) occurred in these experiments, even though the underlying theory was based on motion (approach or avoidance). There are important differences between isometric activation, active movement, and passive movement. During isometric activation, although an active motor process (isometric tension) with an efference copy, the neural indicator of agency, is involved [20], receptors located in the respective muscles and the joints immediately inform the brain about the lack of actual movement. Also, the aforementioned receptors signal to the brain if the arm is moving passively, as the motor command and the corresponding efference copy (agency) are not involved in the process [20]. Furthermore, beyond muscle activation, movement can also become associated with evaluation. The importance of agency is also demonstrated by Van Dessel and colleagues [15]. Thus, an interesting question is whether movement in itself can shape affective evaluation, or motor effort (self-generated movement) is also necessary. To test these ideas, we asked participants to evaluate neutral stimuli during active (generated by own muscle effort) or passive (performed by a machine) flexion and extension of the elbow joint. It was assumed, that (1) both active and passive flexion would be associated with more positive, whereas extension in both conditions would be associated with more negative evaluation, and (2) this effect would be larger for the active motion condition, because it involves muscle effort (agency) that may strengthen the effect, and also because of the self-generated nature of the movement.

## Methods

The study was preregistered prior to data collection. The registry is available at: https://doi.org/10.17605/OSF.IO/NM82V. The statistical analysis and the data are available at: https://osf.io/3fmqv/files/osfstorage.

### Participants

Participants were recruited through an undergraduate course. The target sample size was calculated with the G*Power software v3.1.9.4 [21]. The goal was to obtain 0.95 power to detect a medium effect size (f = 0.25) at the standard 0.05 alpha error probability for the iteration term of a two-factorial (2*3 levels), repeated measures ANOVA. The calculation indicated a minimal sample size of n = 54. The final sample consisted of 56 right-handed participants, with a mean age of 20.2 years (SD = 3.1 yrs, 80% women). Participation was voluntary; students were compensated with partial course credits for taking part in the experiment. Participants signed a written informed consent form before the measurements; the Research Ethics Committee of Eötvös Loránd University approved the experiment (approval number: 2021/505). Data was collected and recorded anonymously, and the recruitment period lasted from December 15, 2021, to November 10, 2022.

### Procedure

**Fig 1:**
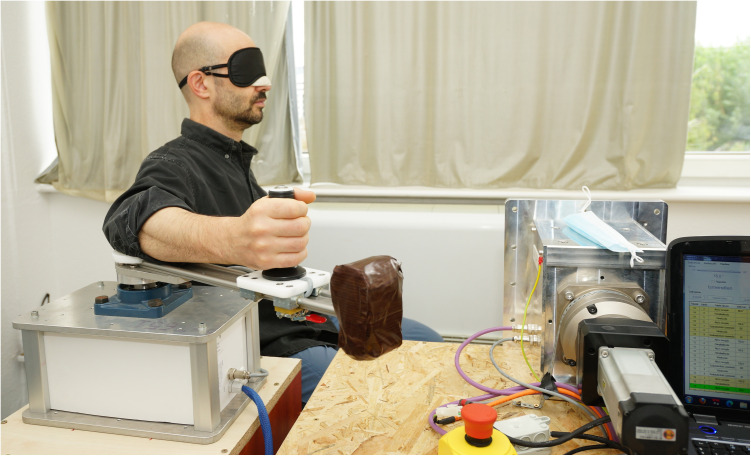
The custom-made motorized device used in the study. The eyes of the participants were not covered in this study.

After arriving at the laboratory, participants read and signed the informed consent form and filled out the questionnaires (demographic data and Positive and Negative Affectivity Schedule, see below). Afterward, they were asked to give baseline preference ratings about 36 Chinese ideographs (adapted from Hull [22]) on an 8-point scale without any experimental manipulation. In the main part of the study, participants had to rate the 36 Chinese ideographs in an active and passive motion block. We used a custom-made motorized device (for further description see [23]) to accurately (+/- 0.1°) move the right (dominant) lower arm and measure the angular position of the right elbow joint (Fig 1). Higher degrees represented a more extended elbow joint. Both the active and passive motion blocks consisted of three movement direction conditions, i.e., 12 trials when the arm moved towards the body (from 150° to 30°), 12 trials when it moved away from the body (from 30° to 150°), and 12 control trials when it remained in a stationary position (at 90°). In the passive motion block, the arm was moved by the motorized device; the duration of movement was 4 seconds. In the active movement block, participants were instructed to move their arm, and the device blocked the motion at the endpoint (proper velocity of the movements was practiced before the trials). The ideographs were presented during movement of the arm for the toward and away conditions. For the stationary condition, the ideographs were presented for 4 seconds after the arm reached 90°. Note, that the only difference between passive and active control (stationary) blocks was how the arm got to the position (by the device or by the participant moving it). However, this block was included in both conditions to keep the design as balanced as possible, and to keep the two-factorial (movement direction and type) statistical design. Participants were asked to evaluate the presented ideograph immediately after the 4-second period on an 8-point scale by pressing the corresponding numerical button on the keyboard. They sat in front of the 1920x1080 pixel resolution screen, approximately 60 cm from it. For the inside condition, the arm not only moved towards the participant but towards the screen (thus the stimuli) too, consistently with the distance regulation approach [8]. The order of the two blocks, that of the three movement conditions within the blocks, as well as the presentation order of the ideographs, were randomized.

### Measurements

#### Preference ratings.

Participants had to rate their preferences on an 8-point Likert scale (“*Please rate the impression the stimulus made on you*”; 1 – *very negative*, 2 – *negative*, 3 – *slightly negative* 4 – *minimally negative*, 5 – *minimally positive*, 6 – *slightly positive*, 7 – *positive*, 8 – *very positive*).

#### State and trait affectivity.

We used the Hungarian version [24] of the Positive and Negative Affectivity Schedule (PANAS) [25] to assess state and trait affectivity of the participants. The questionnaire consists of 20 items rated on a 5-point Likert scale (from “very slightly or not at all” to “extremely”). 10 items belong to the positive affectivity scale (PA; referring to states such as enthusiasm, activity, alertness), and 10 items assess negative affectivity (NA; e.g., distress, nervousness, fear). For the trait version, participants were instructed to fill out the questionnaire based on how they feel most of the time, while for the state version, they were asked to rate their actual status. Internal consistency values (Cronbach’s alpha coefficients) are: .83 for PA_state_, .67 for NA_state_, .85 for PA_trait_, and .84 for NA_trait_.

#### Statistical analysis.

Statistical analysis was conducted using the JASP v0.16.4 software [26]. Baseline ratings’ deviation from neutral was checked with a one-sample t-test with 4.5 as a reference value. To control for the possible individual differences in preferences, the respective baseline ratings were subtracted from the ratings in each trial (i.e., negative values indicate that the preference shifted to the negative direction in the trial). The corrected preference ratings’ deviation from zero (i.e., no difference from the baseline rating) was checked with one-sample t-tests. Associations between baseline preference ratings and questionnaire scores were estimated with Pearson’s correlation. To test our hypotheses, we used two-factorial (2*3) repeated measures analysis of variance (ANCOVA). Motion (active and passive) was represented by one factor, whereas the three movement conditions (toward the body, away from the body, no movement) were represented by another factor. The covariates were PA_trait_ and NA_trait_ for one model, and PA_state_ and NA_state_ for the other; these covariates were included to control for participants’ affective bias caused by their general proneness and actual mood state, respectively. Bayesian ANCOVAs with an identical design were also run (i.e., null models included subject, the respective questionnaire scores, and random slopes for repeated measures factors). In these analyses, JASP’s default prior settings and values (0.5 for r scale fixed effects; 1 for r scale random effects, 0.354 for r scale covariates) were applied; individual comparisons are based on the default t-test with a Cauchy (0, r = 1/sqrt(2)) prior. In Bayesian analysis, a Bayes Factor (BF_10_) larger than 3 was considered to support the alternative hypothesis, whereas BF_10_ < 0.33 was regarded as support for the null hypothesis [27].

## Results

### Explorative analysis

**Fig 2:**
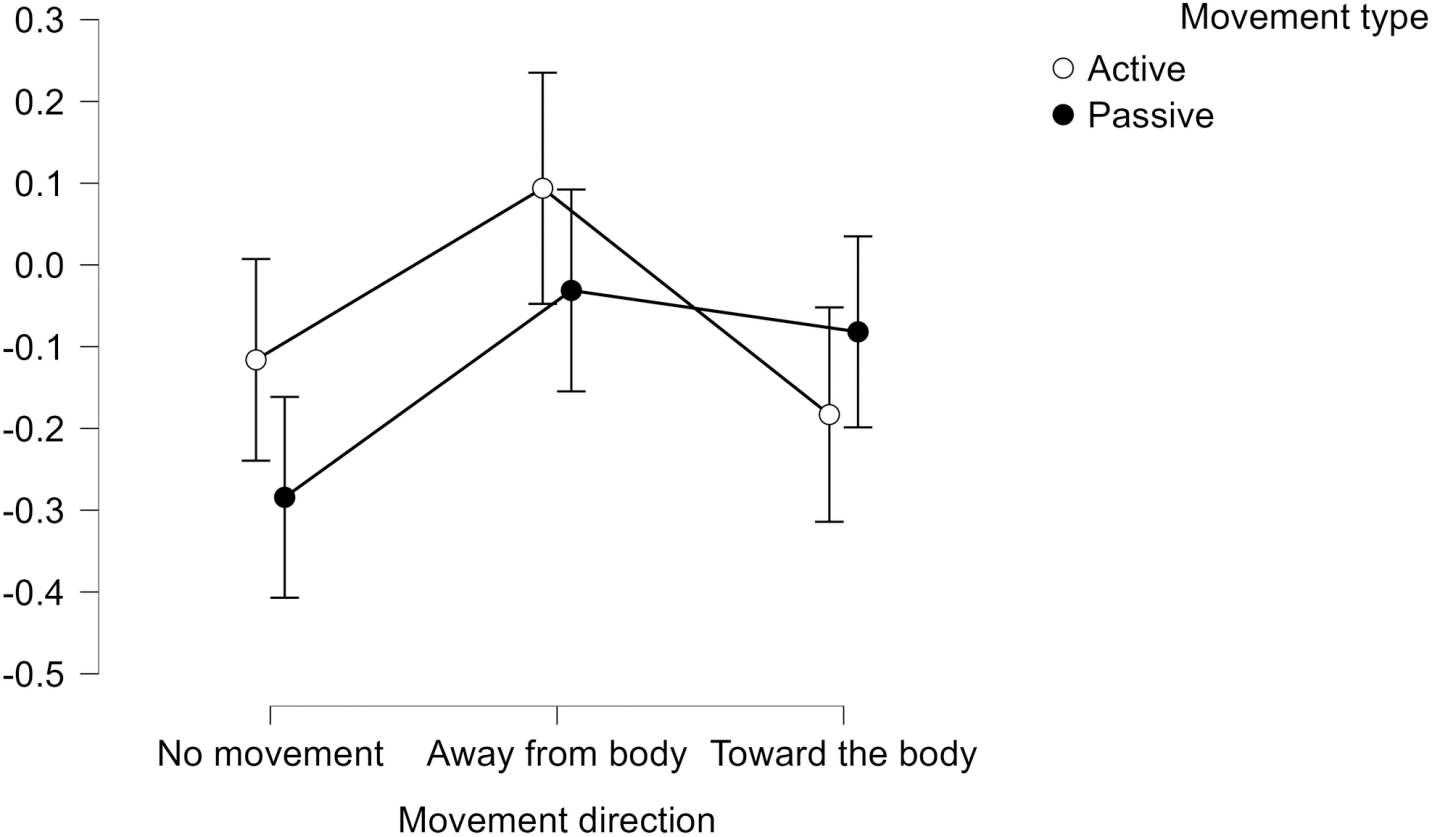
Means and 95% confidence intervals of preference ratings in the different conditions.

One sample t-test indicated that baseline ratings were significantly higher (i.e., more positive) than the neutral value of 4.5 (M = 4.96, SD = .52, t(55) = 6.66, p < .01, d = .89). Descriptive statistics of the corrected preference ratings are presented in Table 1 and Fig 2. Motion toward the body in the active motion condition and no movement in the passive motion condition were significantly smaller than zero, indicating a negative shift in the evaluation relative to the baseline rating.

**Table 1:** Descriptive statistics (M ± SD) of the preference rating in the different experimental conditions, and results of one-sample t-tests checking deviation from zero.

N = 56	M ± SD	T(55); p; Cohen’s d
Active, no movement	−0.12 ± 0.54	−1.60; 0.12; −0.21
Active, toward the body	−0.18 ± 0.56	−2.44; 0.02; −0.33
Active, away from body	0.09 ± 0.52	1.34; 0.20; 0.18
Passive, no movement	−0.28 ± 0.58	−3.67; < 0.01; −0.49
Passive, toward the body	−0.08 ± 0.54	−1.14; 0.26; −0.15
Passive, away from body	−0.03 ± 0.58	−0.40; 0.69; 0.13

Descriptive statistics for the trait and state versions of PA and NA scales are presented in Table 2. Baseline preference ratings showed no significant association with PA_state_ (r = .25, p = .06), NA_state_ (r = −.25, p = .06), PA_trait_ (r = .16, p = .24), and NA_trait_ (r = −.03, p = .84).

**Table 2:** Descriptive statistics of the questionnaire scores.

N = 56	M	SD	min	max
PA state	2.95	0.61	1.40	4.60
NA state	1.43	0.36	1.00	2.40
PA trait	3.41	0.62	1.80	4.60
NA trait	1.77	0.55	1.00	3.30

Note: PA: Positive Affectivity, NA: Negative affectivity.

### Hypothesis testing

Repeated measures ANCOVA with trait questionnaire scores as covariates showed no significant movement direction main effect (*F*(2,106) =.25, *p* = .78, *η*^*2*^ < .01), movement type effect (*F*(1,53) = 1.99, *p* = .16, *η*^*2*^ < .01), and movement direction*movement type interaction (*F*(2,106) =.12, *p* = .89, *η*^*2*^ < .01). In addition, none of the interactions with questionnaire scores were significant. As an explorative analysis, we have conducted the Bayesian analysis of the same model, where *BF*_*10*_ = 3.561 for movement direction, *BF*_*10*_ = .199 for movement type, and *BF*_*10*_ = .913 for motion and condition.

Repeated measures ANCOVA with state questionnaire scores as covariates showed no significant movement direction (*F*(2,106) = 1.70, *p* = .19, *η*^*2*^ < .01), movement type (*F*(1,53) = 2.516, *p* = .119, *η*^*2*^ < .01), and movement type*movement direction interaction (*F*(2,106) =.10, *p* = .91, *η*^*2*^ < .01). Only the movement type x PA_state_ interaction was significant (*F*(1,53) = 4.189, *p* = .05, *η*^*2*^ < .01). As an explorative analysis, we have conducted the Bayesian analysis of the same model, which indicated *BF*_*10*_ = 9.270 for movement direction, *BF*_*10*_ = .222 for movement type, and *BF*_*10*_ = .507 for the interaction term. *Post hoc* tests indicated *BF*_*10*_ = 23.657 for the differences between no movement and away from the body conditions (away from the body condition showing higher mean), *BF*_*10*_ = .18 for no movement *vs* toward the body. The difference between the toward body *vs* away from body conditions was inconclusive (*BF*_*10*_ = 2.461).

## Discussion

In a laboratory experiment with the participation of 56 young individuals, no differences in preference ratings obtained while moving the forearm toward *vs* away from the body were found after controlling for state and trait negative and positive affectivity. Also, no difference between active (muscular) and passive (machine-made) motion was found.

In contrast to the previous ones [1,2,11,12,16–18], our study relied on actual (fore)arm movements, i.e., flexion and extension of the elbow joint. Under such conditions, arm movements toward and away from the body were not characterized by different preference ratings. In addition, the majority of preference ratings did not differ from zero, i.e., arm movements did not lead to a shift in participants’ preferences. Mean ratings in the passive motion control condition and the active motion toward the body condition were significantly smaller than zero, indicating a negative shift in preferences compared to baseline ratings. These findings are not in accordance with results reported in previous studies applying isometric muscle work and thus do not support the idea that approaching and departing forearm movements are associated with a positive and negative evaluative bias, respectively. Although Bayesian analysis indicated a substantial difference between the no movement and away from the body conditions (the latter being more positive than the former), this difference does not support the aforementioned findings either.

There may be more than one explanation for the lack of effect observed in our study. First of all, it is possible that not the specific pattern of muscle activation (i.e., the movement) is important, but the meaning attached to, similar to what was found when investigating the effect of valenced stimuli on motor behaviour [8], or even the effect of movement on detection threshold of negative and positive stimuli [28]. Participants probably did not automatically associate meanings (i.e., approach and avoidance) to elbow flexion and extension. Another explanation is the involvement of actual movements (i.e., isotonic muscle work) and its intensity. Moving the device in the active condition required minimal effort, whereas no muscle work occurred in the passive condition. Isometric effort used in previous studies was not exactly measured; the actual effort was typically described as slight or light for the table-pressing paradigm [1,11,12,16,29]. In studies involving carrying/moving a basket/cart [16–18] the actual effort was also not precisely assessed (and it must have changed during the experiment). However, in comparison to these tasks, muscle effort was probably lighter in our study, which might also explain the lack of differences within conditions. In addition, although trait and state positive and negative affectivity can impact preference ratings, previous studies did not control for these constructs.

Concerning the hypothesis that active motion leads to more marked shifts in preferences than passive motion, Bayesian analysis supported the lack of such a difference. This can be due to the minimal muscle effort needed to actively move the device (see above). It can be concluded that arm motion alone (i.e., without at least a slight muscle effort) is not enough to evoke changes in preference ratings.

An obvious limitation of the current study concerns the student sample, which was not representative of the general population, limiting the generalizability of the findings. The stimulus set we used was evaluated as slightly positive at baseline, which might have affected subsequent ratings. Also, internal consistency of NA_state_ was relatively low.

In conclusion, slight isotonic and passive movements of the forearm do not change affective evaluation of stimuli. Systematic manipulation of the parameters of the study, most importantly the muscle effort needed to move the forearm, and the meaning associated with the motor actions could shed more light on the background of the results.
